# Adherence to a Digital Knee Rehabilitation Platform Among Patients With Knee Osteoarthritis and Anterior Cruciate Ligament Reconstruction in Hong Kong: Qualitative Study

**DOI:** 10.2196/87471

**Published:** 2026-06-26

**Authors:** Nipuna Cooray, Stacie Powell, Mingqian Yu, Michael Tim Yun Ong, Julie Brown

**Affiliations:** 1 Injury Division, Faculty of Medicine and Health, University of New South Wales Sydney, The George Institute for Global Health, Level 8, Health Translation Hub, 55 Botany Street, Randwick, New South Wales, 2031, Australia, 61 2 8052 4300; 2 Department of Orthopaedics & Traumatology, Chinese University of Hong Kong, Hong Kong SAR, China (Hong Kong)

**Keywords:** digital rehabilitation, exercise adherence, knee osteoarthritis, anterior cruciate ligament, thematic analysis, Hong Kong

## Abstract

**Background:**

Exercise therapy is fundamental to rehabilitation for knee osteoarthritis and anterior cruciate ligament (ACL) reconstruction, yet adherence to prescribed exercise typically declines once clinical supervision ends. Digital rehabilitation platforms offer a promising means of supporting sustained exercise adherence, but qualitative evidence on how patients experience these platforms in real-world clinical practice remains limited, particularly in non-Western health care contexts.

**Objective:**

This study aimed to explore how patients with different knee conditions experienced the Healthy Knees digital rehabilitation platform in Hong Kong and to identify the factors shaping their platform engagement and exercise adherence.

**Methods:**

A qualitative design was adopted using reflexive thematic analysis. Fifteen adults (9 with ACL, 6 with osteoarthritis) who had been prescribed the Healthy Knees web-based platform at Prince of Wales Hospital participated in semistructured, in-person interviews (30‐45 min). Interviews were conducted in Cantonese or Mandarin, transcribed verbatim, translated into English, and analyzed inductively. Ethics approval was obtained from the Chinese University of Hong Kong and the University of New South Wales.

**Results:**

Participants were aged 21 to 79 years, with most being male (11/15). Younger participants were predominantly patients with postoperative ACL, while older participants were predominantly patients with preoperative osteoarthritis. Three interrelated themes were identified, collectively describing the fit between the platform and participants’ contexts. Content fit captured the alignment between exercise content and rehabilitation needs; participants across both groups perceived substantial overlap with existing physiotherapy, and content was often mismatched to their recovery stage. Motivational fit captured the alignment between platform support features and motivational needs; pain functioned as both a driver and a deterrent to exercise, and participants ranged from highly self-directed to reliant on external scaffolding, not following a simple age pattern. Access fit captured the alignment between the platform’s delivery mechanism and participants’ technological circumstances; QR code–dependent access, absence of a dedicated mobile app, and display issues created friction that led several participants to migrate to alternative resources, maintaining exercise adherence while abandoning platform engagement.

**Conclusions:**

Adherence to digital knee rehabilitation was shaped by the degree of fit between the platform and users’ contexts across content, motivational, and access dimensions. When access fit failed, participants often substituted alternative exercise resources rather than ceasing exercise entirely, highlighting a distinction between platform engagement and exercise adherence. As the sample’s clinical and demographic characteristics were closely linked, these findings should not be interpreted as diagnostic comparisons between ACL and osteoarthritis populations but as patterns shaped by the recovery phase and age. These findings suggest that digital rehabilitation platforms should incorporate adaptive content aligned with the recovery stage, integrated feedback mechanisms, and reduced access friction to sustain platform engagement within an ecosystem of competing alternatives.

## Introduction

Exercise is widely recognized as a key approach in managing knee osteoarthritis and improving recovery following knee surgeries, such as total knee replacement (TKR) and anterior cruciate ligament (ACL) reconstruction [1]. Therapeutic exercise programs are critical in reducing pain, improving joint function, and enhancing overall quality of life for individuals with knee impairments [2].

Despite the proven benefits of these programs, maintaining exercise for patients remains a significant challenge. Exercise adherence usually declines once clinical supervision tapers off [3]. A decline in exercise can lead to suboptimal recovery outcomes, prolonged rehabilitation periods, and increased health care costs [4,5].

Digital rehabilitation interventions—including web-based platforms and mobile apps—have emerged as promising tools to support sustained exercise adherence beyond the clinic [6]. These platforms can offer flexible access to exercise guidance, structured programs, and remote monitoring capabilities [7]. Yet, the effectiveness of such interventions depends on whether patients engage with and adhere to them in practice [8]. Adherence itself is a complex, multidimensional phenomenon: the World Health Organization has argued for a systems approach that considers socioeconomic, health system, condition-related, therapy-related, and patient-related factors, rather than treating nonadherence as a patient-driven problem [9]. Understanding how digital rehabilitation interventions are experienced in real-world settings—and why users persist with, adapt, or abandon them—is therefore essential for informing intervention design [10].

A growing body of quantitative research has examined digital rehabilitation for knee conditions, with evidence suggesting that digital adjuncts can improve short-term adherence, though benefits may diminish over time [11]. However, qualitative research exploring patients’ lived experiences of digital knee rehabilitation remains limited, particularly in non-Western health care contexts. Existing qualitative work has identified broad themes such as the importance of personalization, usability, and motivational support [6], but it is not well understood how patients with differing clinical and demographic profiles experience digital knee rehabilitation platforms in practice, nor how their experiences shape adherence in practice. There is also limited evidence from contexts such as Hong Kong, where long public hospital waiting times for elective orthopedic surgery [12], significant physiotherapy workforce shortages, and the growing availability of alternative digital health resources create a distinctive landscape for digital rehabilitation adoption.

**Figure 1.**
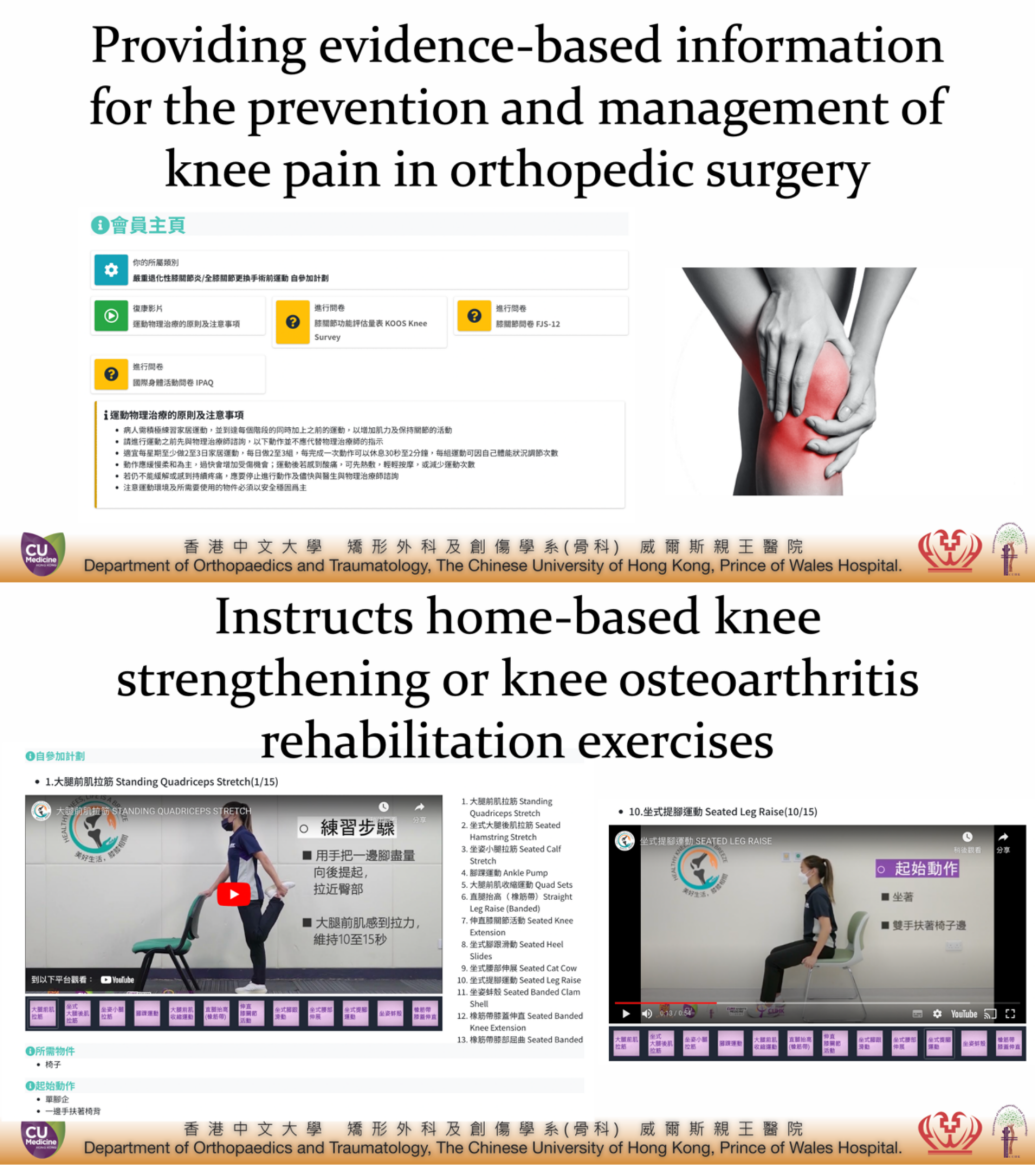
Healthy knees digital intervention.

The Healthy Knees intervention (Figure 1) is a web-based rehabilitation platform developed by the Department of Orthopaedics and Traumatology at the Chinese University of Hong Kong. It provides evidence-based information for the prevention and management of knee pain in orthopedic settings and delivers structured, home-based rehabilitation exercises for patients with knee osteoarthritis and those recovering from ACL surgery. The platform includes video demonstrations of strengthening and mobility exercises, educational resources on knee health, and progress monitoring tools such as patient-reported outcome surveys. The platform uses online questionnaires to monitor at-risk patients. It is accessed via a web browser using a QR code provided by the treating clinician; no dedicated mobile app is available. The platform does not include automated adherence-support features, such as goal-setting tools, personalized feedback, or social comparison functions. It is intended to support remote rehabilitation by improving access to exercise guidance outside conventional in-person physiotherapy services.

In this study, adherence refers to the extent to which participants performed the prescribed exercises after accessing them through the Healthy Knees platform. This encompasses both engaging with the platform to access exercise content and subsequently completing exercises as intended. The study did not capture objective platform usage metrics such as login frequency or session duration; rather, adherence was explored through participants’ self-reported accounts of their exercise behavior in qualitative interviews. The term “adherence” is used consistently throughout this paper; where a distinction is necessary, “platform engagement” refers specifically to interaction with the digital platform, while “exercise adherence” refers to the performance of prescribed exercises.

This study qualitatively examines adherence to the Healthy Knees digital rehabilitation platform among individuals with different knee conditions in Hong Kong. The aim is to explore how patients experience the platform in real-world clinical practice and to identify the factors that shape sustained engagement and exercise behavior across different clinical profiles.

## Methods

### Overview

This qualitative study used reflexive thematic analysis following the 6-phase process described by Braun and Clarke [13,14]. Trustworthiness was addressed using the strategies outlined by Nowell et al [15], including prolonged engagement with the data and peer debriefing. The study was planned and designed by NC, SP, JB, and MTYO. NC and SP trained MY, who conducted the interviews. The researchers, except for MTYO, who was the consulting clinician, had no prior relationships with the participants. The study was reported using the COREQ (Consolidated Criteria for Reporting Qualitative Research). The study was conducted at Prince of Wales Hospital in Hong Kong.

The research team brought diverse disciplinary perspectives to the study. NC is a digital health researcher with no clinical relationship with the participants. SP contributed qualitative research expertise and had no prior involvement with the Healthy Knees platform or its users. JB provided senior research oversight for the project. MTYO, the consulting orthopedic clinician, introduced patients to the Healthy Knees platform as part of routine clinical care and was involved in study design but did not participate in recruitment, conduct interviews, or contribute to the analysis. MY, who conducted the interviews, was trained by NC and SP and had no prior relationship with participants. Participants were existing users of the Healthy Knees platform who were invited to participate by MY during their clinic visit. The team acknowledges that participants’ awareness of MTYO involvement in their clinical care may have influenced their willingness to participate or the candor of their responses. This should be considered when interpreting the findings.

### Participant Selection and Recruitment

Participants were selected using a convenience sampling strategy from patients at Prince of Wales Hospital who had been introduced to the Healthy Knees app by author MO as their consulting clinician. Participants were recruited during their clinic visits. The sample included individuals diagnosed with knee osteoarthritis, those awaiting ACL surgery, and those who had undergone ACL surgery. Inclusion criteria focused on patients who had been introduced to the Healthy Knees app. No explicit exclusion criteria were defined for this study.

### Data Collection

Data were collected through in-person, semistructured interviews. A total of 15 interviews were conducted, each lasting around 30 to 45 minutes. The interviews were designed to identify the participants’ experiences with the Healthy Knees app.

The research team developed a semistructured interview guide (Multimedia Appendix 1) to facilitate the interviews. The guide began with questions to collect participant demographic and clinical background information, followed by open-ended questions exploring participants’ experiences with the Healthy Knees platform, their perceptions of the exercises, barriers and facilitators to adherence, motivations for exercise, and suggestions for platform improvement. Probing questions were used to elicit detailed responses. Prior to data collection, the interview guide was pilot-tested within the research team to ensure clarity and relevance. In practice, the interviewer used the guide flexibly, adapting the order and phrasing of questions in response to participants’ accounts, consistent with a semistructured approach.

All interviews were conducted in Cantonese or Mandarin by MY and were audio-recorded. The audio recordings were transcribed verbatim in the original language. The first transcript was translated into English by MY. The remaining transcripts were translated by a professional translation service at Guangdong University of Foreign Studies, a university recognized for its expertise in translation studies and accredited by the International Conference of University Institutes of Translators and Interpreters. No formal back-translation or independent verification of translations was conducted; this is acknowledged as a limitation.

### Data Analysis

Data analysis followed the 6-phase process of reflexive thematic analysis [13,14]. NC familiarized with the data by reading the transcripts multiple times and then generated initial codes by identifying key patterns, concepts, and recurring ideas. These codes were grouped into broader categories in the search for potential themes, which were subsequently reviewed, refined, and named to ensure they accurately represented the data and were distinct from one another. Throughout this process, NC, SP, and JB engaged in recurring discussions to critically examine the developing codes and themes, ensuring they were grounded in the data and reflected the range of participant experiences. Where individual accounts diverged from emerging patterns, these were examined closely to assess whether theme boundaries required refinement. During the final phase of analysis, the team identified an overarching pattern connecting the 3 themes, which was developed inductively rather than applied from a pre-existing theoretical framework. The coding and analysis were carried out manually without using software tools. A completed COREQ checklist is provided as Checklist 1.

### Ethical Considerations

Ethical approval for the study was obtained from the Chinese University of Hong Kong (2023.29), and external approval was registered with the University of New South Wales (iRECS5785) ethics committees. Informed consent was obtained from all participants through a signed written consent form before participation. Participants were informed of the study’s purpose, that participation was voluntary, and that they could withdraw at any time without affecting their clinical care. All interview transcripts were deidentified prior to analysis, with participants assigned alphanumeric codes (eg, P01) in place of identifying information, and stored securely on password-protected institutional servers accessible only to the research team. Participants did not receive any financial or other compensation for taking part in the study.

This project was funded by the Chinese University of Hong Kong-The George Institute Joint Collaborative Research Fund.

## Results

### Participants

Fifteen participants took part in the study (Table 1). The sample comprised 9 participants with ACL conditions and 6 with knee osteoarthritis. Most were male (11/15). Notably, diagnosis, age, and surgical stage were closely intertwined in this sample: participants with ACL conditions were predominantly younger (age range 21‐52 y) and postoperative (8/9), while participants with osteoarthritis were predominantly older (age range 63‐79 y) and preoperative (5/6). This overlap means that differences observed between the 2 groups cannot be attributed to diagnosis alone and may reflect age, surgical stage, or their combination. Throughout the “Results” section, participants are described by their experiential profile (eg, “younger, postoperative participants”) with the associated diagnosis noted in parentheses.

**Table 1.** Participant demographics^a^.

Characteristic	ACL^b^ (n=9)	Osteoarthritis (n=6)	Total (n=15)
Surgery status, n			
Presurgery	1	5	6
Postsurgery	8	1	9
Sex, n			
Male	8	3	11
Female	1	3	4
Age (y), range	21‐52	63‐79	21‐79

a Detailed clinical variables, such as Kellgren-Lawrence grade for knee osteoarthritis and time since surgery for postoperative participants, were not systematically collected in this study.

b ACL: anterior cruciate ligament.

Analysis of the interview data identified 3 interrelated themes that collectively describe the fit between the Healthy Knees platform and participants’ contexts. These themes—content fit, motivational fit, and access fit—capture the dimensions along which the platform aligned or misaligned with participants’ rehabilitation needs, motivational profiles, and technological circumstances. Across the 3 themes, adherence was shaped not by any single factor but by the degree of alignment across all 3 dimensions.

### Content Fit: Alignment Between Platform Exercises and Rehabilitation Needs

This theme captures the extent to which the Healthy Knees exercise content matched participants’ perceived rehabilitation needs at their particular stage of recovery. Three patterns emerged: perceived redundancy with existing physiotherapy, temporal mismatch between platform content and recovery stage, and difficulty calibrating between exercises and physical capacity.

### Perceived Redundancy With Existing Care

Across both groups, a recurring finding was that participants perceived substantial overlap between the Healthy Knees content and the exercises they were already receiving through physiotherapy or other sources. This perception reduced the platform’s added value and contributed to disengagement:

*I saw that the exercises were similar to those in physical therapy, so I didn’t persist.*

[P01, male, 76 years, osteoarthritis, preoperative]

*They’re the same by 70% to 80%. The physiotherapy exercises consist of stretching routines.*

[P09, male, 45 years, ACL, preoperative]

*I am not interested because they’re very basic.*

[P04, male, 26 years, ACL, postoperative]

Even among participants who found the content acceptable, the platform was often seen as confirming what they already knew rather than offering new guidance. One older participant noted:

*I’ve received these conservative treatments and know how to perform these movements. However, I just do them depending on my own abilities.*

[P05, male, 77 years, osteoarthritis, preoperative]

Similarly, 1 postoperative participant who had progressed well under the guidance of her personal coach found the platform content comparable to what she was already doing (P08, female, 63 years, osteoarthritis, post-TKR). This pattern was cross-cutting, appearing in younger postoperative and older preoperative participants alike, suggesting that redundancy with existing care was a common barrier regardless of age or diagnosis.

### Temporal Mismatch Between Content and Recovery Stage

Among younger, postoperative participants (predominantly ACL), a distinct pattern emerged in which the platform content was seen as appropriate for a particular recovery stage—but not for the stage the participant was at when they accessed it.

*These exercises are suitable for those who need rehabilitation training immediately after surgery. However, I was not at the rehabilitation stage when I accessed this platform.*

[P13, male, 38 years, ACL, approximately 1 year postoperative]

One participant discovered the platform 6 months after surgery, by which time she had already resumed her preinjury physical activities daily and found the early-stage content no longer relevant: “I searched for related videos on YouTube...there weren’t many videos in this respect on YouTube and were not so detailed as this platform” (P14, female, 21 years, ACL, postoperative). Another attempted the exercises immediately after injury but found them too painful:

*I tried to follow your videos to do home exercises immediately after starting the rehabilitation at home. However, doing those movements was so painful that I failed*.

[P02, male, 52 years, ACL, postoperative]

A notable counter-case was P07 (male, 35 years, ACL, postoperative), who reviewed later-stage content and found it genuinely challenging:

*The jumping with the BOSU ball and the single-leg jump are challenging. But I think they are movements at the final stage of the rehabilitation program on your platform. I have not reached that stage currently.*

This suggests that the platform does contain progressive content, but participants who entered at the wrong recovery stage were unlikely to discover or reach it.

### Difficulty Calibration

Perceptions of exercise difficulty diverged along the age and surgical-stage lines in this sample. Younger, postoperative participants (predominantly ACL) consistently found the exercises to be below their capacity and preinjury activity levels:

*Your platform is good. Maybe as an athlete I might think the exercises on the platform are easy, but they are still useful for the elderly.*

[P04, male, 26 years, ACL, postoperative]

*Your exercises are too basic for them, but are adequate for me.*

[P06, male, ACL, postoperative]

Older, preoperative participants (predominantly osteoarthritis) more frequently reported that specific exercises exceeded their physical capabilities:

*This movement is challenging for me, but if I find it beneficial, I will strive to do it even despite difficulties. However, the third and fourth movements are beyond my capacity.*

[P05, male, 77 years, osteoarthritis, preoperative]

*There were a few exercises that I couldn’t do at all, such as bending my feet, stilting my feet.*

[P01, male, 76 years, osteoarthritis, preoperative]

However, this pattern was not universal. P11 (male, 79 years, osteoarthritis, preoperative) found all exercises within his capacity, and P08 (female, 63 years, osteoarthritis, post-TKR) noted that exercises initially difficult became manageable over time: “At the beginning, I found some movements difficult, but now I can do them.” One participant’s experience highlighted a potential safety concern: after stretching for one to two minutes rather than the recommended 10 to 30 seconds, he experienced adverse effects: “I’ve done them for a week and stopped after my muscles hurt. Maybe I didn’t follow their recommended duration” (P09, male, 45 years, ACL, preoperative). This suggests that without adequate guidance on exercise dosage, some users may inadvertently overexert.

### Motivational Fit: Alignment Between Platform Support and Motivational Needs

This theme captures how participants’ motivational profiles interacted with the platform’s support features (or lack thereof) to shape adherence. Two patterns emerged: the complex role of pain as both a driver and deterrent of exercise behavior, and the spectrum between self-directed motivation and reliance on external scaffolding.

### Pain as a Double-Edged Motivational Driver

Across both groups, pain emerged not simply as a barrier to exercise but as a motivational force operating in 2 opposing directions. For some participants, the presence of pain drove continued engagement; for others, it triggered disengagement.

Among older, preoperative participants (predominantly osteoarthritis), pain served primarily as a motivator through fear of functional decline. Despite finding exercises difficult, P01 (male, 76 years, osteoarthritis, preoperative) persisted because he feared deterioration: “Not really fond of exercises, but if I don’t exercise, my physical function may deteriorate soon.” P11 (male, 79 years, osteoarthritis, preoperative) continued exercising because exercises directly alleviated his symptoms: “After doing these exercises, the pains of thighs can be alleviated to some extent.” P05 (male, 77 years, osteoarthritis, preoperative) adopted a selective approach, performing exercises that were tolerable and skipping those that caused excessive discomfort:

*I will skip the movements that are totally beyond my capacity. But I definitely will do these stretches.*

Among younger, postoperative participants (predominantly ACL), the relationship was more paradoxical. P06 (male, ACL, postoperative) explicitly described reduced pain as a reason to stop exercising: “When I feel the therapeutic effect without pain, I tend to skip exercises, otherwise I’m motivated to do more exercises.” In this case, the absence of pain—which would typically signal recovery—actually undermined adherence by removing the perceived need for continued exercise. By contrast, P09 (male, 45 years, ACL, preoperative) stopped exercising because pain from overexertion deterred him: “I’ve done them for a week and stopped after my muscles hurt.”

The Healthy Knees platform did not include features to help participants interpret pain in the context of their recovery—for instance, distinguishing expected postexercise discomfort from signals to reduce intensity. In the absence of such guidance, participants developed their own heuristics, with some persisting through pain and others withdrawing from exercise entirely.

### Self-Direction Versus External Scaffolding

Participants varied considerably in the degree to which they could sustain exercise independently, and this variation did not follow a simple age or diagnosis pattern.

Several participants described strong internal motivation. P04 (male, 26 years, ACL, postoperative) reported no need for external prompts due to his athletic background. P10 (female, 74 years, osteoarthritis, preoperative) exercised 45 minutes to 1 hour daily, combining exercises sourced from a physiotherapy platform (HA Go) with a community exercise group led by a retired coach:

*I think at least if you have some kind of this kind of platform or introduce some patient, at least they have the knowledge to know and then they have the freedom whether to follow is up to their decision.*

Her case challenges any simple association between older age and low self-direction.

Others, however, described difficulty maintaining routines without external structure. P03 (male, 21 years, ACL, postoperative) was candid:

*I don’t think I have that high level of self-consciousness. I will have greater drive to perform workouts with companions than alone.*

P06 (male, ACL, postoperative) attributed his nonadherence to “laziness,” and P01 (male, 76 years, osteoarthritis, preoperative) described a cycle of initial effort followed by abandonment: “I can do it for 10, 20 times, and then I gave up.” One participant (P08, female, 63 years, osteoarthritis, post-TKR) resolved this by building a structured external routine—weekly sessions with a coach supplemented by independent practice at home—effectively creating her own scaffolding outside the platform.

Two younger, postoperative participants independently raised the idea that progress tracking and feedback features could bridge the gap between self-direction and external support. P15 (male, 34 years, ACL, postoperative) described this in detail:

*If I have the record about what exercises I do at what time, the results, are they good or bad, or easy to do? Then I will have more motivation. With something to feedback.*

P13 (male, 38 years, ACL, postoperative) similarly suggested staged content with expected milestones so users could gauge their progress. While this pattern was observed in only 2 participants, both arrived at a convergent suggestion: that the platform’s lack of feedback mechanisms left a motivational gap that neither purely self-directed nor externally prompted approaches could fill.

### Access Fit: Alignment Between Platform Design and Users’ Technological Context

This theme captures how the design and delivery mechanism of the Healthy Knees platform interacted with participants’ technological circumstances to shape engagement. Two patterns emerged: access barriers created by the platform’s delivery format and competition from alternative exercise resources that participants found easier to use.

### Platform Access Barriers

The Healthy Knees platform is accessed via a web browser using a QR code provided on a printed pamphlet during a clinic visit. It has no dedicated mobile app, no saved login, and no bookmark prompt. Several participants described how this access mechanism created friction that disrupted or prevented engagement.

The most direct account came from P08 (female, 63 years, osteoarthritis, post-TKR), who lost the pamphlet and was unable to return to the platform:

*Sometimes it was impossible to access your platform without scanning the QR code … I lost the pamphlet; without the QR code, I couldn’t log into the website.*

Rather than abandoning exercise, she switched to YouTube videos and sessions with her coach.

P01 (male, 76 years, osteoarthritis, preoperative) faced a more fundamental barrier: he did not have a mobile data plan, relying on a pay-as-you-go card, and could only access the platform via home Wi-Fi. A nurse assisted him with initial access during his clinic visit, but sustained engagement required connectivity he did not routinely have:

*My phone doesn’t have internet access. I didn’t sign a service contract.*

P03 (male, 21 years, ACL, postoperative)—a younger participant with no digital literacy difficulties—identified the format itself as the problem and suggested a solution:

*Maybe it would be more convenient to change your platform into an app. Users can view video as soon as they access the app.*

He noted this would also benefit older users: "Some elderly users may not be able to find the QR code each time, but can always check videos on the app with one click.”

One participant reported a technical display issue that compromised usability:

*I have an issue with your website on my mobile phone. Only the latter half is visible; the upper portion remains hidden … It’s an iPhone issue.*

[P06, male, ACL, postoperative]

These barriers were not exclusively a function of age or digital literacy. While older participants faced additional challenges around connectivity and device familiarity, younger participants also encountered friction from the platform’s web-based, QR code–dependent format. The common thread was that the access mechanism placed the burden of re-entry on the user, and when that burden exceeded a threshold—whether through a lost pamphlet, absent connectivity, or a display bug—engagement ceased.

### Competition With Alternative Exercise Resources

A distinctive finding was that when the platform’s access mechanism failed or when content was perceived as insufficient, participants did not simply stop exercising. Instead, many migrated to alternative resources they found more accessible or better suited to their needs.

The most frequently cited alternative was HA Go, a mobile app provided by the Hospital Authority that offers exercise content with daily reminders. P05 (male, 77 years, osteoarthritis, preoperative) described it as functionally comparable to Healthy Knees: “HA Go app has similar functions to your platform and can remind users to do exercises every day.” P10 (female, 74 years, osteoarthritis, preoperative) used HA Go as her primary exercise resource and had not engaged with Healthy Knees at all, supplementing the app with a community exercise group:

*They gave me also another platform that is belonged to the physiotherapist. And then I follow that kind of exercise. I think there’s some improvement for me.*

P11 (male, 79 years, osteoarthritis, preoperative) similarly used HA Go rather than Healthy Knees for his daily exercise routine, responding to its SMS text messaging reminders.

YouTube was another common alternative. P14 (female, 21 years, ACL, postoperative) searched for ACL recovery exercises on YouTube during her home rehabilitation, though she noted the content was “not so detailed as this platform.” P08 (female, 63 years, osteoarthritis, post-TKR) turned to YouTube after losing access to Healthy Knees. P01 (male, 76 years, osteoarthritis, preoperative) took a different approach entirely, purchasing a stretching board and devising his own exercise routine based on prior physiotherapy knowledge.

This pattern of substitution has important implications. It suggests that the participants in this study were not, on the whole, lacking in motivation to exercise—rather, the Healthy Knees platform was competing for their engagement within an ecosystem of alternative resources, some of which offered lower access friction (HA Go’s app format, YouTube’s familiarity) or more personalized guidance (personal coaches, community groups). Nonengagement with the platform, in these cases, did not equate to nonadherence to exercise.

Table 2 summarizes the 3 themes describing the fit between the Healthy Knees platform and participants’ rehabilitation contexts.

**Table 2.** Summary of themes describing the fit between the Healthy Knees platform and participants’ rehabilitation contexts.

Theme	Description
Content fit	The degree to which the platform’s exercise content matched participants’ rehabilitation needs, including perceived redundancy with existing physiotherapy, temporal mismatch between content and recovery stage, and difficulty calibration relative to physical capacity.
Motivational fit	The alignment between the platform’s motivational support features and participants’ needs, including the dual role of pain as both a driver and deterrent of exercise, and the spectrum between self-directed motivation and reliance on external scaffolding.
Access fit	The alignment between the platform’s delivery mechanism and participants’ technological circumstances, including access barriers created by the QR code–dependent web format and competition from alternative exercise resources such as HA Go and YouTube.

Across the 3 themes, a common pattern emerged inductively during analysis: adherence to the Healthy Knees platform depended on the degree of fit between the platform and the participant’s context across content, motivational, and access dimensions. When fit was adequate across all 3 dimensions, participants maintained both platform engagement and exercise adherence. When any single dimension was misaligned—whether through content that did not match the recovery stage, insufficient motivational scaffolding, or access friction—adherence was disrupted. Notably, the consequences of misfit differed by dimension: content and motivational misfit tended to reduce exercise adherence overall, while access misfit more often redirected exercise behavior toward alternative resources, disrupting platform engagement while preserving exercise adherence. Furthermore, fit was not static: participants’ alignment with the platform shifted over the course of their recovery, with content that was initially appropriate becoming redundant or insufficient, and motivational needs evolving as pain and functional capacity changed. These patterns, including the temporal dimension of fit and its implications for platform design, are developed further in the Discussion section.

## Discussion

### Principal Findings

**Figure 2.**
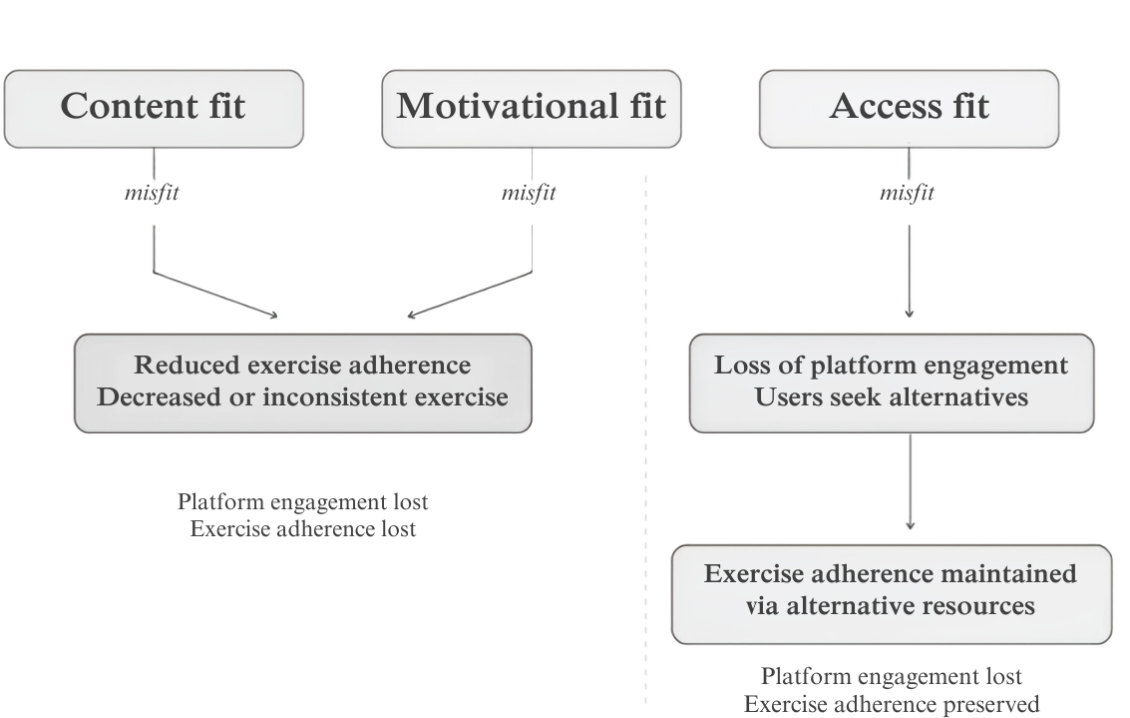
Relationship between the 3 fit dimensions and divergent adherence patterns.

This qualitative study explored adherence to the Healthy Knees digital knee rehabilitation platform among 15 participants in Hong Kong. Three themes were identified—content fit, motivational fit, and access fit—which together describe the alignment between the platform and participants’ rehabilitation needs, motivational profiles, and technological circumstances. The central finding is that adherence was not determined by any single factor but by the degree of fit across all 3 dimensions. When fit was adequate across content, motivation, and access, participants maintained both platform engagement and exercise adherence. When any single dimension was misaligned, adherence was disrupted—but the nature of that disruption differed: content and motivational misfit tended to reduce exercise adherence overall, while access misfit more commonly redirected exercise behavior toward alternative resources, preserving exercise adherence while disrupting platform engagement. These predominant patterns are depicted in Figure 2.

### Fit as an Organizing Framework for Understanding Digital Rehabilitation Adherence

The concept of fit that emerged from this analysis resonates with established adherence frameworks while offering a more specific account of how adherence operates in digital rehabilitation contexts. The World Health Organization’s multidimensional adherence model identifies 5 interacting dimensions—socioeconomic, health system, condition-related, therapy-related, and patient-related factors—and emphasizes that nonadherence is a systemic issue rather than a patient-driven problem [9]. Our findings align with this principle: participants’ nonadherence reflected mismatches in the intervention design, health care delivery context, and access mechanisms, not simply a lack of motivation. The fit framework extends this perspective by specifying the dimensions most salient in a digital rehabilitation context and by revealing that different types of misfits produce qualitatively different patterns of nonadherence.

Our findings can also be situated within the Capability, Opportunity, and Motivation Behavior (COM-B) model of behavior change [16], which posits that capability, opportunity, and motivation must all be present for a behavior to occur. Content fit maps broadly onto capability—participants needed exercises that were appropriate to their physical capacity and recovery stage. Motivational fit maps onto motivation—participants varied in the degree to which they could sustain exercise without external scaffolding. Access fit maps onto opportunity—the platform’s delivery mechanism either enabled or constrained the possibility of engagement. Consistent with previous applications of COM-B to rehabilitation contexts [17], our findings suggest that interventions addressing only 1 component—for example, providing exercise content without motivational support or accessible delivery—are unlikely to sustain adherence. However, the fit framework adds a temporal dimension that COM-B does not explicitly address: fit is not static but shifts as users progress through recovery, meaning that a platform well-suited to a participant at one stage may become misaligned at another.

### Content Fit and the Challenge of Perceived Redundancy

A striking cross-cutting finding was that participants across both groups perceived the Healthy Knees content as substantially overlapping with exercises they were already receiving through in-person physiotherapy or other digital resources. This finding suggests that providing evidence-based content alone may be insufficient to drive engagement; rather, as the person-based approach to digital intervention development argues, content must offer perceived added value from the user’s perspective to sustain use [18]. The temporal mismatch finding—in which participants accessed the platform at a recovery stage for which the content was either too advanced or no longer relevant—has important design implications. Previous work on digital rehabilitation has emphasized the importance of tailoring exercise content to the individual [19], but our data suggest that tailoring must account not only for diagnosis and physical capacity but also for the timing of platform introduction relative to the patient’s rehabilitation trajectory. The experience of participants who discovered the platform months after surgery, when its early-stage content was no longer relevant, illustrates how the window of optimal content fit can be missed entirely. Conversely, participants who attempted exercises immediately postinjury found the content too demanding, suggesting that premature introduction can be equally problematic.

The safety concern raised by one participant, who overexerted during stretching exercises and experienced adverse effects, highlights the limitations of a platform that provides exercise demonstrations without guidance on dosage or adaptation. Digital rehabilitation platforms that lack real-time feedback or clear dosage parameters may inadvertently place responsibility for safe exercise progression on the user, which is consistent with concerns raised in the wider literature on unsupervised home-based exercise [20].

### Motivational Fit and the Dual Role of Pain

The finding that pain operated simultaneously as a motivator and a deterrent adds nuance to existing understandings of adherence in musculoskeletal rehabilitation. Previous research has identified pain as a barrier to exercise adherence [21], but our findings suggest a more complex dynamic in which the presence or absence of pain served as an implicit indicator of need. For some participants, feeling pain signaled that exercise was necessary; for others, the absence of pain removed the perceived rationale for continued effort. This paradox has been observed in medication adherence research, where symptom improvement is a well-documented trigger for discontinuation [9], but it has received less attention in the digital rehabilitation literature.

The spectrum between self-directed motivation and reliance on external scaffolding also challenges simple demographic assumptions. Some of the most self-directed exercisers in the sample were older preoperative participants, while some younger postoperative participants described significant difficulty maintaining routines without companions or external prompts. This finding is consistent with research suggesting that health literacy, prior exercise experience, and self-efficacy may be more important predictors of sustained engagement than age alone [22]. The convergent suggestion from 2 participants that progress tracking and feedback features could support adherence aligns with evidence that self-monitoring is among the most effective behavior change techniques in digital health interventions [23].

### Access Fit and the Ecosystem of Alternative Resources

The finding that participants migrated to alternative exercise resources rather than ceasing exercise entirely is perhaps the most distinctive contribution of this study. Recent work on engagement with digital health interventions has drawn a useful distinction between microengagement—interaction with the digital tool itself—and macroengagement—engagement with the target health behavior [8,24]. Our findings illustrate this distinction empirically: several participants maintained macroengagement (exercise adherence) while abandoning microengagement (platform engagement), substituting YouTube videos, the HA Go app, personal coaches, or self-devised routines.

This pattern of substitution reflects the competitive landscape in which digital rehabilitation platforms operate. In Hong Kong, the Hospital Authority’s HA Go app provides a widely available, app-based alternative with built-in reminder features. YouTube offers a vast library of rehabilitation exercise content. Personal coaches and community exercise groups provide social support and accountability. The Healthy Knees platform, delivered via a QR code–dependent web browser with no dedicated app, no saved login, and no automated reminders, faced a significant access disadvantage relative to these alternatives. This is consistent with broader evidence that the usability and convenience of digital health tools are critical determinants of sustained engagement, particularly when users have readily available alternatives [25].

### The Hong Kong Context

Several features of the Hong Kong health care system shaped the adherence patterns observed in this study. Waiting times for elective orthopedic surgery in the public system can extend to several years [12], meaning that participants with preoperative osteoarthritis in this study were managing their condition over prolonged periods with limited physiotherapy access. Participants described physiotherapy sessions conducted at long intervals that felt insufficient, with limited opportunities for 1-on-1 attention. This context makes digital rehabilitation particularly relevant as a means of bridging gaps in formal care—but it also means the platform must sustain engagement over long waiting periods, a challenge that the current static content model is unlikely to meet.

The cost differential between public and private physiotherapy in Hong Kong is substantial. Participants in this study were drawn from a public hospital where physiotherapy resources were described as limited. Digital platforms offer a potential solution to this resource constraint, but our findings suggest that a platform perceived as duplicating rather than extending existing care will not be adopted, regardless of its clinical quality.

While these health care system factors are specific to Hong Kong, several of the adherence patterns observed in this study may extend to other contexts. Context-specific findings include the reliance on a QR code–based web platform as the primary access mechanism and the availability of HA Go as a readily accessible alternative provided by the Hospital Authority. However, the challenge of perceived redundancy with existing physiotherapy, the temporal mismatch between static platform content and evolving rehabilitation needs, and the tendency for users to substitute alternative resources when access friction is high are likely relevant wherever digital rehabilitation platforms operate alongside established care pathways.

### Strengths and Limitations

This study has several strengths. To the authors’ knowledge, it is among the first qualitative studies to explore digital knee rehabilitation adherence in Hong Kong, providing context-specific insights into a health care system with distinctive resource constraints and cultural characteristics. The postimplementation design captured real-world experiences of participants who had used the platform in practice, rather than hypothetical responses to a prototype. The inclusion of both ACL and osteoarthritis participants allowed comparison across different experiential profiles, revealing both cross-cutting and group-specific patterns.

Several limitations must be acknowledged. Diagnosis, age, and surgical stage were closely intertwined in this sample: participants with ACL were predominantly younger and postoperative, while participants with osteoarthritis were predominantly older and preoperative. Consequently, differences observed between groups cannot be attributed to diagnosis alone, and may reflect age, perioperative stage, or their combination. Detailed clinical descriptors—such as osteoarthritis severity grading, time since surgery, or concomitant injuries—were not systematically collected, which limits the degree to which findings can be linked to specific clinical characteristics. Future research should include such variables to enable more precise stratification.

The study used a convenience sample drawn from a single hospital, potentially limiting transferability, although there is no reason to expect patients at this site to differ substantially from those at other public hospitals in Hong Kong. The platform did not capture objective usage data (eg, login frequency, session duration), so all engagement information is based on participants’ self-reported accounts. Transcripts were translated from Cantonese or Mandarin into English, which may have introduced minor shifts in meaning despite efforts to ensure accuracy through the use of a professional translation service. Data collection did not pursue theoretical saturation, and with 15 participants, some perspectives may not have been captured. While our sample included diverse demographic and clinical profiles, future research with larger and more varied samples is needed to explore additional viewpoints.

### Conclusions

Adherence to digital knee rehabilitation is best understood not as a property of the individual or the platform, but as a product of the ongoing fit between the 2 across content, motivational, and access dimensions. This fit is dynamic, shifting as users progress through recovery. Digital rehabilitation platforms that offer static content through high-friction access mechanisms risk losing users to more accessible alternatives—without necessarily losing them to inactivity. Designing for sustained fit, particularly through adaptive content, integrated feedback, and reduced access friction, may be more effective than targeting adherence as an isolated behavior. Future research should test this framework longitudinally and with objective usage data to identify when and how fit breaks down across recovery trajectories.

## Supplementary material

10.2196/87471 Multimedia Appendix 1 Interview discussion guide.

10.2196/87471 Checklist 1 COREQ checklist.

## Abbreviations

ACL: anterior cruciate ligament
COM-B: Capability, Opportunity, and Motivation Behavior
COREQ: Consolidated Criteria for Reporting Qualitative Research
TKR: total knee replacement
