# Supplementary material for: Adherence to a Digital Knee Rehabilitation Platform Among Patients With Knee Osteoarthritis and Anterior Cruciate Ligament Reconstruction in Hong Kong: Qualitative Study
Source: JMIR Rehabil Assist Technol. 2026 Jun 26;13:e87471. doi: 10.2196/87471 (PMC13309063; doi:10.2196/87471)
Supplement: Multimedia Appendix 1 [file rehab-v13-e87471-s001.docx]

Discussion Guide

Introduction:

a. Welcome the participant and explain the purpose of the interview.

b. Obtain informed consent and ensure confidentiality.

Participant Background:

a. Age, occupation, and relevant medical history (e.g., type of knee surgery/condition, any complications)

b. Previous experience with exercise programs, physical therapy, or rehabilitation

Experience with the Healthy Knees Program:

a. How did you learn about the program?

b. What were your expectations before starting the program?

c. How easy or difficult was it to access and navigate the program?

Adherence to the Program:

a. How often did you use the program and for how long?

- Prompts

b. Did you follow the program consistently? If not, why?

c. Were there specific exercises or features that were particularly helpful or challenging?

- Prompts -if yes, what aspects of the program you liked the most (knowing you have professional assistance/monitored by the doctor, easy-to-follow exercise guides, physically able exercises, reminders/emails, self-monitoring aspects, ongoing motivational support)

Barriers to Adherence:

a. What obstacles or challenges did you face while using the program?

b. Did you experience any technical difficulties or issues with the program's design?

c. How did your physical condition, motivation, or other personal factors affect your adherence?

- Prompts – diminishing motivation, exercises too hard, exercise advice unclear/complex, no professional support integrated, no self-monitoring

Facilitators to Adherence:

1. What aspects of the program encouraged or helped you to follow it consistently?

b. Did you receive any support from healthcare professionals, family, or friends?

c. Were there any incentives, rewards, or feedback mechanisms that motivated you?

- Prompts - knowing you have professional assistance/monitored by the doctor, easy-to-follow exercise guides, physically able exercises, reminders/emails, self-monitoring aspects, ongoing motivational support

Program Outcomes:

a. How has your knee recovery progressed since starting the program?

b. Did the program meet your expectations? Why or why not?

c. Would you recommend the program to others in a similar situation?

Suggestions for Improvement:

a. What improvements or changes would you suggest for the program?

b. How could the program better support users in adhering to the exercise plan?

c. Are there any additional features or resources that would have been helpful?

Conclusion:

a. Thank the participant for their time and valuable insights.

b. Explain how their input will be used and any follow-up steps.
