# Supplementary material for: Adherence to a Digital Knee Rehabilitation Platform Among Patients With Knee Osteoarthritis and Anterior Cruciate Ligament Reconstruction in Hong Kong: Qualitative Study
Source: JMIR Rehabil Assist Technol. 2026 Jun 26;13:e87471. doi: 10.2196/87471 (PMC13309063; doi:10.2196/87471)
Supplement: Checklist 1 [file rehab-v13-e87471-s002.docx]

**Multimedia Appendix 2**

**COREQ (COnsolidated criteria for REporting Qualitative research) Checklist**

*Developed from: Tong A, Sainsbury P, Craig J. Consolidated criteria for reporting qualitative research (COREQ): a 32-item checklist for interviews and focus groups. International Journal for Quality in Health Care. 2007;19(6):349–357.*

| **No.** | **Topic** | **Guide questions/description** | **Reported on page/section** |
| --- | --- | --- | --- |
| **Domain 1: Research team and reflexivity** | | | |
| ***Personal characteristics*** | | | |
| 1 | **Interviewer/facilitator** | Which author/s conducted the interview or focus group? | MY conducted all interviews (Methods, Study Design and Data Collection sections) |
| 2 | **Credentials** | What were the researcher’s credentials? | NC: digital health researcher; SP: qualitative research expertise; JB: senior researcher; MO: consulting orthopaedic clinician; MY: trained interviewer (Methods, Reflexivity paragraph) |
| 3 | **Occupation** | What was their occupation at the time of the study? | NC, SP, JB: university researchers; MO: orthopaedic clinician at Prince of Wales Hospital (Methods, Reflexivity paragraph) |
| 4 | **Gender** | Was the researcher male or female? | Not reported |
| 5 | **Experience and training** | What experience or training did the researcher have? | MY was trained by NC and SP prior to data collection (Methods, Reflexivity paragraph) |
| ***Relationship with participants*** | | | |
| 6 | **Relationship established** | Was a relationship established prior to study commencement? | MY had no prior relationship with participants. MO was the consulting clinician (Methods, Reflexivity paragraph) |
| 7 | **Participant knowledge of the interviewer** | What did the participants know about the researcher? | Participants were informed of the study purpose at the start of each interview (Methods, Data Collection) |
| 8 | **Interviewer characteristics** | What characteristics were reported about the interviewer/facilitator? | MY was bilingual (Cantonese/Mandarin/English) and had no clinical relationship with participants (Methods, Reflexivity paragraph and Data Collection) |
| **Domain 2: Study design** | | | |
| ***Theoretical framework*** | | | |
| 9 | **Methodological orientation** | What methodological orientation was stated to underpin the study? | Reflexive thematic analysis following Braun and Clarke (2006, 2019). Trustworthiness addressed using Nowell et al. (2017) (Methods, Study Design and Data Analysis) |
| ***Participant selection*** | | | |
| 10 | **Sampling** | How were participants selected? | Convenience sampling from patients at Prince of Wales Hospital who had been introduced to the Healthy Knees platform (Methods, Participant Selection and Recruitment) |
| 11 | **Method of approach** | How were participants approached? | Invited to participate by MY during their clinic visit (Methods, Reflexivity paragraph) |
| 12 | **Sample size** | How many participants were in the study? | 15 participants (Results, Participants section and Table 1) |
| 13 | **Non-participation** | How many people refused to participate or dropped out? Reasons? | Not reported |
| ***Setting*** | | | |
| 14 | **Setting of data collection** | Where was the data collected? | In-person at Prince of Wales Hospital, Hong Kong (Methods, Study Design and Data Collection) |
| 15 | **Presence of non-participants** | Was anyone else present besides the participants and researchers? | Not reported |
| 16 | **Description of sample** | What are the important characteristics of the sample? | Age, sex, diagnosis (ACL/OA), and surgical status reported in Table 1 and Participants section (Results) |
| ***Data collection*** | | | |
| 17 | **Interview guide** | Were questions, prompts, guides provided by the authors? | A semi-structured interview guide was developed, pilot-tested, and used flexibly. Provided as Multimedia Appendix 1 (Methods, Data Collection) |
| 18 | **Repeat interviews** | Were repeat interviews carried out? | No; single interviews only |
| 19 | **Audio/visual recording** | Did the research use audio or visual recording to collect the data? | All interviews were audio-recorded (Methods, Data Collection) |
| 20 | **Field notes** | Were field notes made during and/or after the interview? | Not reported |
| 21 | **Duration** | What was the duration of the interviews? | 30–45 minutes each (Methods, Data Collection) |
| 22 | **Data saturation** | Was data saturation discussed? | Saturation was not pursued; acknowledged as a limitation (Discussion, Limitations) |
| 23 | **Transcripts returned** | Were transcripts returned to participants for comment? | No member checking was conducted |
| **Domain 3: Analysis and findings** | | | |
| ***Data analysis*** | | | |
| 24 | **Number of data coders** | How many data coders coded the data? | Initial coding was conducted by NC, with themes reviewed and refined through discussion with JB and SP (Methods, Data Analysis) |
| 25 | **Description of the coding tree** | Did authors provide a description of the coding tree? | The process of generating codes and grouping them into candidate themes is described. An audit trail of highlighted notes with initial codes was maintained (Methods, Data Analysis) |
| 26 | **Derivation of themes** | Were themes identified in advance or derived from the data? | Themes were derived inductively from the data (Methods, Data Analysis) |
| 27 | **Software** | What software, if applicable, was used to manage the data? | Coding and analysis were carried out manually without software tools (Methods, Data Analysis) |
| 28 | **Participant checking** | Did participants provide feedback on the findings? | No member checking was conducted |
| ***Reporting*** | | | |
| 29 | **Quotations presented** | Were participant quotations presented to illustrate the themes? | Yes; direct quotations with participant identifiers (age, sex, diagnosis, surgical status) are presented throughout the Results |
| 30 | **Data and findings consistent** | Was there consistency between the data presented and the findings? | Themes are supported by multiple quotations from participants across both clinical groups (Results) |
| 31 | **Clarity of major themes** | Were major themes clearly presented in the findings? | Three themes (content fit, motivational fit, access fit) are clearly defined with subthemes and a summary table (Results, Table 2) |
| 32 | **Clarity of minor themes** | Is there a description of diverse cases or discussion of minor themes? | Divergent cases are identified and discussed within each theme (Results). The team attended to cases that did not conform to emerging patterns (Methods, Data Analysis) |
